# Handling missing values in patient-reported outcome data in the presence of intercurrent events

**DOI:** 10.1186/s12874-025-02510-8

**Published:** 2025-03-01

**Authors:** Doranne Thomassen, Satrajit Roychoudhury, Cecilie Delphin Amdal, Dries Reynders, Jammbe Z. Musoro, Willi Sauerbrei, Els Goetghebeur, Saskia le Cessie, on behalf of SISAQOL-IMI Work Package 3, Doranne Thomassen, Satrajit Roychoudhury, Cecilie Delphin Amdal, Dries Reynders, Jammbe Z. Musoro, Willi Sauerbrei, Els Goetghebeur, Saskia le Cessie, Rajesh Kamalakar, Kavita Sail, Ethan Basch, Jaap Reijneveld, Christoph Gerlinger, Ahu Alanya, Gerhard Rumpold, Maxime Sasseville, Jennifer Black, Geert Molenberghs, Khadija Rantell, Michael Schlichting, Antoine Regnault, David Ness, Silene ten Seldam, Tove Ragna Reksten, Anja Schiel, Ragnhild Sorum Falk, Alicyn Campbell, Joseph C. Cappelleri, Alexander Russell-Smith, Melanie Calvert, Samantha Cruz Rivera, Olalekan Lee Aiyegbusi, Limin Liu, Kelly Van Lancker, Claudia Rutherford, Vishal Bhatnagar, Ting-Yu Chen, Mallorie Fiero, Paul Kluetz

**Affiliations:** 1 Department of Biomedical Data Sciences, Leiden University Medical Center, Leiden, The Netherlands; 2 Pfizer Inc, New York, NY USA; 3 Research Support Services, Oslo University Hospital, Oslo, Norway; 4 Department of Oncology, Oslo University Hospital, Oslo, Norway; 5 Department of Applied Mathematics, Computer Science and Statistics, Ghent University, Ghent, Belgium; 6 European Organisation for Research and Treatment of Cancer (EORTC) Headquarters, Brussels, Belgium; 7 Institute of Medical Biometry and Statistics, Faculty of Medicine and Medical Center - University of Freiburg, Freiburg, Germany; 8 Department of Clinical Epidemiology, Leiden University Medical Center, Leiden, The Netherlands

**Keywords:** Patient-reported outcomes, Missing data, Multiple imputation, Estimand, Intercurrent event, Repeated measurements

## Abstract

**Introduction:**

As patient-reported outcomes (PROs) are increasingly used in the evaluation of medical treatments, it is important that PROs are carefully analyzed and interpreted. This may be challenging due to substantial missing values. The missingness in PROs is often closely related to patients’ disease status. In that case, using observed information about intercurrent events (ICEs) such as disease progression and death will improve the handling of missing PRO data. Therefore, the aim of this study was to develop imputation models for repeated PRO measurements that leverage information about ICEs.

**Methods:**

We assumed a setting in which missing PRO measurements are missing at random given observed measurements, as well as the occurrence and timing of ICEs, and potentially other (baseline or time-varying) covariates. We then showed how these missingness assumptions can be translated into concrete imputation models that also account for a longitudinal data structure. The resulting models were applied to impute anonymized PRO data from a single-arm clinical trial in patients with advanced lung cancer.

**Results:**

In our trial example, accounting for death and other ICEs in the imputation of missing data led to lower estimated mean health-related quality of life (while alive) compared to an available case analysis and a naive linear mixed model imputation.

**Conclusion:**

Information about the timing and occurrence of ICEs contribute to a more plausible handling of missing PRO data. To account for ICE information when handling missing PROs, the missing data model should be separated from the analysis model.

**Supplementary Information:**

The online version contains supplementary material available at 10.1186/s12874-025-02510-8.

## Introduction

Patient-reported outcomes (PROs) are increasingly used to provide evidence of efficacy and tolerability of treatments in clinical trials, including oncology [1–4]. As with any other trial outcome, it is important that PROs are carefully analyzed and interpreted, in line with a clear research aim. However, structured approaches to analyze PRO data were found to be lacking in many oncology studies [5–8]. Previous studies have shown how a PRO-related research question may be translated into a well-defined estimand using the recent framework proposed by ICH E9 (R1) [9], and which corresponding statistical methods may be used for estimation [10, 11]. However, little attention has been paid to the alignment of the missing PRO data strategy with the PRO estimand in a study [12–16].

Defining the estimand in a study requires specifying the treatment, the population, the variable of interest, the population-level summary, and a strategy for handling intercurrent events (ICEs) [9, 11]. ICEs refer to events that occur in a trial after the start of treatment and affect the interpretation or existence of measurements of interest. Examples of ICEs are treatment discontinuation and death. Strategies for handling ICEs mentioned in the estimand framework include a while-the-ICE-has-not-occurred strategy, a treatment policy strategy, a hypothetical strategy, a composite variable strategy, and a principal stratum strategy [9, 11]. The estimand framework distinguishes a strategy for handling missing data from the chosen strategy to address intercurrent events [9]. The chosen ICE strategy partly determines which data are required to estimate the estimand and which are considered missing if not collected.

Methods to address missing data should respect the chosen ICE strategy for a coherent analysis and interpretation of results. PROs are often measured at several time points, with the aim of estimating or comparing the mean (change in) PRO value at (a) time point(s) of interest. The traditional way of handling missing longitudinal data is to use (generalized) linear mixed models or mixed models for repeated measures (MMRM) in the analysis [17, 18]. However, such analyses may not align with the chosen ICE strategy. For example, after a patient has died, their PRO data do not exist and are not generally considered missing [9]. Analyses using mixed models assume that missing data are missing at random (MAR) conditional on the model variables [19, 20]. Implicitly, at any time point, the PRO values of those who have died will be considered missing at random and the model will handle these ‘missing’ PRO values based on the observed PRO values in those who are still alive at that time. Therefore, estimated means directly derived from a mixed model correspond to a hypothetical strategy to handle death, where interest is in the PRO in a hypothetical world where patients do not die during the study. This strategy is often applied implicitly but may not always align with the research aim [11].

Additionally, there is a relation between ICEs and the occurrence of missing PRO data. ICEs may cause PRO data to be unavailable: when a patient experiences disease progression, they may be too ill to fill out a questionnaire. On the other hand, the occurrence and timing of ICEs may be correlated with the value of missing PRO measurements. It has been shown that, on average, the health-related quality of life (HRQoL) of patients with cancer deteriorates after disease progression [21, 22] and in the weeks leading up to death [23–28]. As a result, observed information about ICEs may be utilized for a better handling of missing PRO data.

In this SISAQOL-IMI [29] study, we therefore aimed to evaluate imputation methods for missing PRO data that

1. take into account information about the occurrence and timing of intercurrent events;
2. are suitable for repeated measurements data;
3. can be aligned with an estimand of choice; and
4. do not impute PROs after death.

We illustrated our methods using anonymized data from a single-arm clinical trial in patients with advanced lung cancer.

## Methods to handle missing PRO data

### Study setting

Suppose that a trial is analyzed where a PRO was scored on a numerical scale. This PRO was scheduled to be measured at baseline and at one or more predefined time points after baseline. We denote with ${Y}_{ij}$ the PRO reported by patient $i\in \left\{1,\dots ,n\right\}$ at timepoint $j\in \left\{1,\dots ,m\right\}$ where $j=1$ at baseline. Besides the PROs ${Y}_{ij}$, baseline variables ${X}_{i}$ were recorded for each patient $i$, and possibly time-varying variables ${Z}_{ij}$. Additionally, it was recorded when patients passed away during the study, when they were lost to follow-up and when they experienced non-terminal ICEs such as discontinuation of protocol treatment. We assume that interest is in estimating the mean (or mean change in) PRO at one or several time points after baseline (e.g., for nonrandomized studies), or in differences between the means (or mean changes) in different treatment groups (e.g., for randomized studies).

### Estimand and data required for estimation

As mentioned previously, defining an estimand requires specification of one or more strategies to address each ICE, respectively. The selected ICE strategies partly determine the data needed to estimate the estimand of interest and the data that are considered missing if not observed.

When addressing death, a terminal ICE, a *while-alive* strategy may be deemed most appropriate, especially when the study has a descriptive aim [11, 30], as PRO values after death do not exist. An example of a while-alive estimand is the mean PRO at a time point of interest in those who are still alive at that time point, which should be combined with an estimate of the probability to survive until that timepoint. For most other ICE strategies, PRO values after death are not relevant either. In a *composite variable* strategy, a composite endpoint is defined that includes both the PRO and death (e.g., the EQ-5D summary index score or Quality-Adjusted Life Years). A *principal stratum* strategy addresses death by estimating a contrast in PROs between treatment arms in the (counterfactual) subset of patients who would not have died in any of the treatment arms in a study. A *treatment policy* strategy cannot be applied to terminal ICEs such as death, since values for a PRO do not exist after a terminal ICE [9].

The estimation of a *hypothetical* estimand for death considers PRO values in a hypothetical world where patients do not die during the study. By definition, these values are not available after a patient has died in the observed world. But even before death, it is difficult to determine which PRO values in the observed world would be the same in the hypothetical world where death does not occur. For example, in the weeks leading up to death due to cancer, HRQoL tends to deteriorate [24]. In a hypothetical world without death, such a deterioration may not take place. Therefore, observed PRO values in the weeks before a patient’s death may not be suitable for the estimation of a hypothetical estimand for death. Furthermore, there may be settings in which a hypothetical world without death but with disease progression is difficult to imagine.

For non-terminal ICEs, a treatment policy strategy (reflecting the intention-to-treat principle) requires PRO data after the ICE has occurred and this will be considered missing if not collected. A while-the-ICE-has-not-occurred estimand (e.g., while on treatment) and a composite estimand are estimated based on PRO data before the ICE (and information about the ICE itself, for the composite outcome). The data required for estimation of a principal stratum estimand depend on the corresponding subgroup of interest.

Some non-terminal ICEs are defined by a conscious action or intervention, for instance, treatment switching or the addition of rescue medication. For such intervention ICEs, a hypothetical world in which the intervention was not applied might be better imaginable than for ICEs that are not interventions. Methods to estimate hypothetical estimands for intervention ICEs have been described in the literature recently, with links to imputation methods and causal inference techniques [12, 31]. Overall, it is important to clearly define the hypothetical world targeted by a hypothetical estimand and to assess whether observed data are sufficiently in line with this hypothetical world for estimation. Any extrapolation from the observed world to an unobserved, hypothetical world requires untestable assumptions about the similarity of the two worlds.

### Notation to describe the occurrence and timing of ICEs

To include information about the occurrence and timing of ICEs in methods to address missing PRO data, this information must first be encoded. For a schematic representation of the notation introduced below, please see Figure S1, Online Resource 1. Let the variable $${D}_{i}\in \{\text{0,1}\}$$ indicate whether patient $$i$$ died during the study, where $${S}_{i}$$ denotes their corresponding survival time $$({D}_{i}=1)$$ or the time at which they were censored for survival $$\left({D}_{i}=0\right)$$. Further, we suppose that $$K$$ different types of non-terminal ICE could be observed (for example, treatment discontinuation due to toxicity, disease progression), each event denoted by $${E}_{k},k\in \{1,\dots , K\}$$. Analogous to the variables describing survival, the binary variable $${E}_{ik}\in \{\text{0,1}\}$$ denotes whether $${E}_{k}$$ occurred for patient $$i$$ during the study. If $${E}_{k}$$ was indeed observed for patient $$i$$ before the end of their follow-up (i.e., when $${E}_{ik}$$ $$=1$$), $${T}_{ik}$$ denotes the time at which ICE $${E}_{k}$$ was observed for patient $$i$$. If patient $$i$$ did not experience $${E}_{k}$$ during their follow-up (i.e., when $${E}_{ik}=0)$$, then $${T}_{ik}$$ denotes the time at which follow-up for ICE $${E}_{k}$$ ended for patient $$i$$. For example, for disease progression, $${T}_{ik}$$ refers to the time of the last assessment of the disease status. In other words, $${T}_{ik}$$ is an event time if $${E}_{k}$$ is observed, and a censoring time if $${E}_{k}$$ is not observed.

Based on this information, we define the following time-varying variables for each patient $i$:

- ${s}_{ij}$denotes the difference in time units between the survival time ${S}_{i}$ and the current timepoint $j$. As there are no observations after death or censoring for overall survival, ${s}_{ij}$ is nonnegative.
- Analogously, ${t}_{ijk}$ denotes the current ‘time-distance to non-terminal ICE ${E}_{k}$ for patient $i$’: the difference in time units between ${T}_{ik}$ and the current timepoint $j$. The difference ${t}_{ijk}$ is positive when timepoint $j$ is before ${T}_{ik}$, ${t}_{ijk}=0$ at ${T}_{ik}$, and ${t}_{ijk}$ is negative after ${T}_{ik}$.
- ${E}_{ijk}$ indicates whether ICE ${E}_{k}$ has yet been observed at timepoint $j$. For the terminal ICE death, such an indicator is redundant as there are no observations after death.

As a result, each patient is viewed on several timescales at once: the follow-up time that starts at baseline, as well as a timescale relative to each ICE ${E}_{k}$ and death, respectively (Figure S1, Online Resource 1).

### Missing data assumptions

Often, not all PRO scores are recorded at each of the required time points, leading to missing PRO data. We assume that when a patient did not report their outcome at one timepoint, they may still have reported their outcome at future timepoints (unless, of course, death had occurred).

Information on the occurrence and timing of ICEs may make a MAR assumption conditional on observed information more plausible. Therefore, missing PRO measurements at timepoint $j$ are assumed to be MAR conditional on the timepoint $j$, the PRO ${Y}_{i{j}^{*}}$ for other relevant timepoints ${j}^{*}\ne j$, the occurrence and timing of ICEs (${E}_{ik}$, ${E}_{ijk}$, ${t}_{ijk}$, ${D}_{i}$, ${s}_{ij}$), treatment (if applicable), any relevant baseline covariates ${X}_{i}$ and possibly time-varying covariates ${Z}_{ij}$. As ICEs may occur after the missed measurement, this means we assume that future events provide information about what the missed measurement may have been. After death, PRO measurements are not considered missing as they are nonexistent. If an ICE has not occurred during the study, the time of its occurrence may be right-censored at the end of the study. We assume noninformative censoring for simplicity in our examples but methods to account for informative censoring (such as reweighting [32]) can be combined with the missing PRO data strategies outlined in this paper.

### Imputation of missing PRO data

It is important that the chosen method to address missing PRO data accounts for the longitudinal structure of the PRO data, and that it is informed by the occurrence and timing of ICEs, which may be observed after the missing PRO was planned to be measured. For this reason, likelihood-based methods where a mixed model is used for the implicit imputation and analysis of PROs at the same time do not suffice. While the information on (future) ICEs is informative for the missing values, conditioning on future values in an analysis model is inappropriate as this will not target the estimand of interest and yield uninterpretable estimates of the treatment effect. Therefore, as a way to take ICE information into account, missing PRO values should be handled using a missing data model that is separate from the substantive analysis model.

To deal with missing PRO measurements in accordance with our MAR assumptions, we distinguish two types of methods: reweighting methods by the inverse probability of missingness (IPW) and multiple imputation (MI) methods for the missing outcomes [19]. Kurland and Heagerty have shown that IPW methods can be combined with generalized estimating equations (GEE) to estimate and perform inference on marginal means of the (change in) PRO over time under a MAR assumption conditional on the time until death [30]. To obtain such marginal means only in those still alive, an independence working correlation should be specified for the GEE [30]. Recently, Haug et al. have also proposed IPW weights for missing HRQoL data that account for time of death and combined these weights with a machine learning model to estimate average HRQoL over time [33]. These IPW techniques can be extended to include all variables in our MAR assumption described above in the estimation of the IPW weights [32, 34, 35].

For MI, it has been recommended that imputation models are compatible with the intended analysis model, that is, that they include at least all variables from the analysis model as well as their relation presumed in the analysis model (e.g., interactions) [36]. As the PROs in our case are numerical and longitudinal, we assume that the analysis model will be some mixed model or GEE (depending on the estimand) where the outcome is modelled as a (flexible) function of the timepoint $j$, treatment, the outcome at other time points, and potentially covariates. The imputation model would then include these variables, as well as the ICE-related variables in our MAR assumption.

Where possible, we recommend modelling the time-from-ICE variables ${t}_{ijk}$ and ${s}_{ij}$ flexibly while imputing missing PRO values, for example with splines, to allow for nonlinear relationships with the PRO (e.g., a drop in HRQoL in the final weeks before death). Another choice to make is whether to let the value of ${t}_{ijk}$ decrease indefinitely after ${T}_{ik}$ or to keep it constant from some time point, for example, when there are almost no patients with data after ICE${E}_{k}$. We strongly recommend including interactions between the time variables for the ICE (${t}_{ijk}$,${s}_{ij}$) and their corresponding event/censoring indicators (${E}_{ik}$,${D}_{i}$), to model the relationship between the PRO and an event time differently from the relationship between the PRO and a censoring time. If the relation between the PRO and ${t}_{ijk}$ is expected to be different before and after ICE ${E}_{k}$ occurs, interactions between ${t}_{ijk}$ and the time-varying event indicator ${E}_{ijk}$ can also be included. Of course, including splines and interactions in an imputation model requires a sufficiently large dataset and may not always be feasible.

Besides information about ICEs, PROs observed in the same participant at other time points are also likely informative for missing PRO measurements [37]. We distinguish two approaches to include this information in imputation, following Huque et al. [38]. The first option is a cross-sectional approach where, for each missing PRO measurement, PRO measurements at other relevant time points are included explicitly as variables in the imputation model (longitudinal data in wide format). Alternatively, a multilevel approach may be used, where all PRO measurements are treated as one outcome variable with a within- and between-subject correlation (multilevel data structure) and possibly a relationship with time (longitudinal data in long format). Huque et al. showed in a simulation-based comparison that cross-sectional approaches using standard software implementations often performed well [38]. However, the cross-sectional approach requires the variables to be imputed if missing to be the same for every participant. When the number of time points at which PROs are to be imputed differs between participants (for example, to avoid imputation after death), multilevel approaches may be better suited.

For the multilevel MI approach, software implementations in R [39] exist to model the joint distribution of incomplete variables by iterating through univariate models for each incomplete variable conditional on the other variables (fully conditional specification, ‘MI-FCS’) [40]. Within FCS, we noted two types of approaches in the literature for multilevel data. In the first approach, mixed models (i.e., models with random effects) are used as the univariate conditional models (e.g., ‘2l.norm’, in the mice package [41]). The second approach includes using flexible generalized additive models as the univariate conditional models combined with a clustered bootstrap instead of a simple bootstrap to approximate the distribution to impute from (‘aregImpute’ function in the Hmisc package [42]). While marginal means from a (linear) mixed model imply a hypothetical estimand for death, mixed models can be used to impute *individual* missing PRO values in those patients who are still alive. It has been shown that mixed models have a subject-specific interpretation in the population currently alive [43].

## Illustration in a single-arm lung cancer trial

The implementation of several of the methods mentioned was illustrated and compared in real clinical trial data.

### Description of the trial

We illustrated our methods in PRO data from a single-arm, multicenter phase 2 trial [44] evaluating a new anticancer drug in patients with late-stage lung cancer. PRO data were available for 834 patients. Patient-reported HRQoL data, measured with the EORTC QLQ-C30 global QoL scale [45, 46], were included as a secondary endpoint, with scale scores ranging from 0 (representing the worst) to 100 (best possible score). Henceforth, we will refer to this specific PRO as ‘QoL’.

While on trial medication, participants were asked to report on QoL on the first day of protocol treatment and every three weeks while on protocol treatment. We therefore refer to the timing of QoL measurements with their tri-weekly cycle number and baseline is defined here as the first day of cycle 1. After cycle 10 (30 weeks), the study protocol allowed for completion of the questionnaire on the first day of alternate cycles (i.e., every six weeks).

Three types of ICEs were recorded in the data: progression of disease (PD, ${E}_{1}$), treatment discontinuation (TD, ${E}_{2}$), and death. Death was observed in 576 (66%) patients. The Kaplan–Meier estimate of median survival time was 21.7 months [95% CI 19.8–24.2]. The probability of survival was estimated to be 67% [95% CI 64%-71%] at one year and 47% [95% CI 43%-50%] at two years after the start of protocol treatment. PRO data were available until less than 1 month before death for 140 patients (24% of observed deaths), and less than 3 months before death for 315 patients (55%). PRO measurements were mostly available until shortly before censoring of the survival time: the median [IQR] time between the last available PRO and censoring was 1.59 [0.62–16.24] months.

**Fig. 1:**
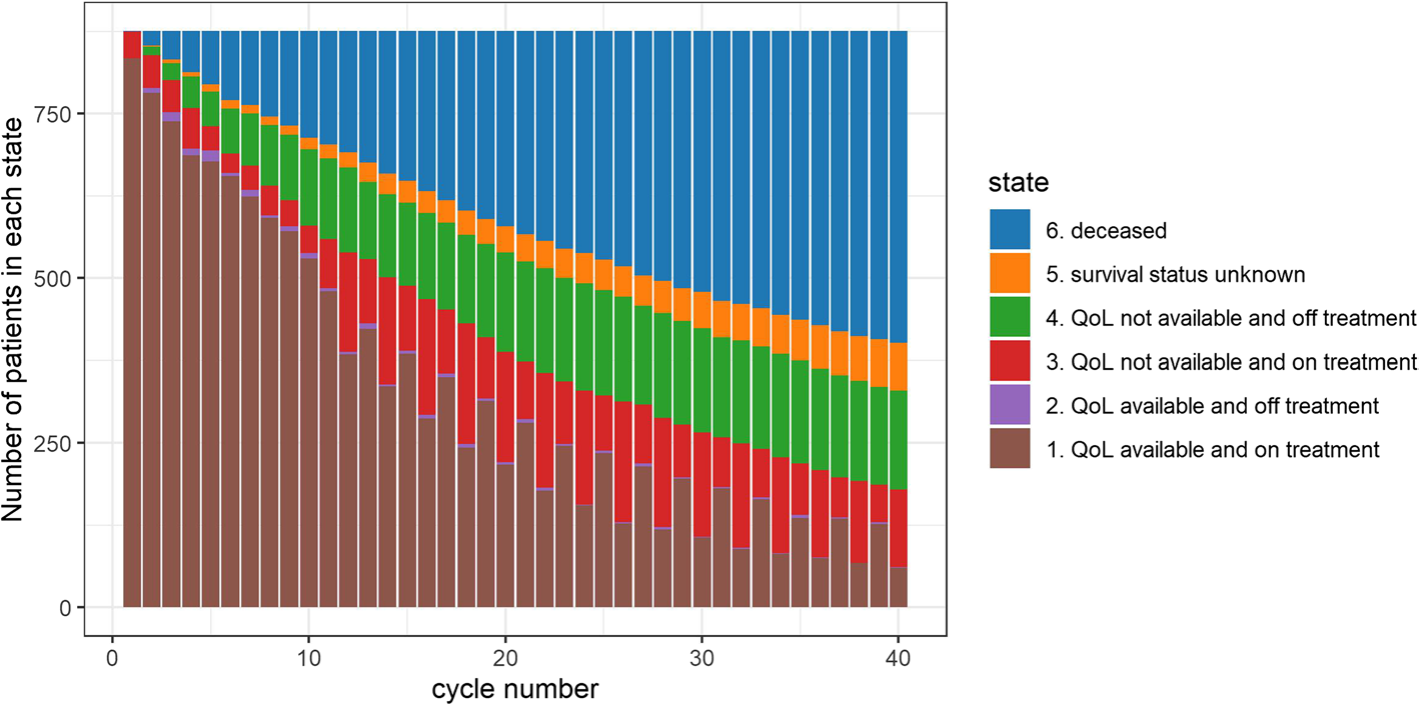
Availability of global QoL scores over time, in relation to treatment and survival status. Note that by design of the study, almost no global QoL scores were reported after discontinuation of protocol treatment. As a result, there are no, to very few patients in state 2 at each cycle. Also note that fluctuations in data availability while on treatment (state 1) reflect the study design: after cycle 10, the study protocol allowed for completion of questionnaires at alternate cycles

Although disease progression was often followed by the discontinuation of treatment, the physician and patient could decide together when to stop the treatment. PROs were collected until PD for most patients in whom PD was recorded (*n* = 648) and there was a substantial number of PROs recorded after PD. After PD, 345 patients continued the trial medication for at least one month and 241 continued for 3 months or more. Upon discontinuation of trial medication, patients were asked to complete one final questionnaire, after which QoL data collection was stopped, while follow-up for overall survival continued. Most patients (89%) reported PROs at the time of treatment discontinuation. Eight patients had two PRO measurements post-discontinuation, and one patient had three PRO-measurements after TD. The trial sponsor anonymized the measurements before they were shared with us. Figure 1 summarizes the availability of QoL data over time in the trial data. A more elaborate description of the anonymized data can be found in a previous case study [11].

### Estimand

In this single-arm trial setting, our analysis aim was to describe HRQoL over time in the study population. Our endpoint of interest was the absolute numerical value of the global QoL score as reported by each patient, at each cycle. The mean global QoL score at cycles 1 through 40 was chosen as the population-level summary. As the trial participants were patients with cancer in an advanced disease stage, many deaths were observed during the study.

The research objective was to estimate the mean global QoL score from the start of study medication up to treatment cycle 40 or death, in the reality where PD and TD occur as they did in the study. Because HRQoL after death is not meaningful, and a hypothetical situation without deaths was far removed from reality in this case, we opted for a while-alive strategy to address death [11]. For cycles 1–40, the mean QoL was estimated at each cycle, within only those patients who were still alive at that cycle, accompanied by an estimate of the survival probability up to that cycle. For the other intercurrent events (PD and TD), a treatment policy strategy was applied. Estimation of this estimand required QoL measurements from each participant for cycle $j=1,\dots ,40$, or until their death if death occurred earlier, irrespective of PD or TD. Where such measurements were not available, they were considered missing data.

### Missing data imputation

#### Variables and coding

We stored the QoL data ${Y}_{ij}$ in long format where each study participant had several rows of data, one row for each predefined QoL measurement cycle $j$. When a patient died or was censored for overall survival, the patient had no rows in the data after this event. As survival times ${S}_{i}$ differed between individuals, the number of rows in the data varied between patients.

Each data row contained a patient identifier $i$, the number of the measurement cycle $j$, and the corresponding QoL measurement ${Y}_{ij}$ (possibly missing). The row also contained time-constant variables ${E}_{ik}$, ${T}_{ik}$, ${D}_{i}$, and ${S}_{i}$; as well as time-varying variables ${E}_{ijk}$, ${t}_{ijk}$, ${s}_{ij}$. Here, $k\in \{\text{1,2}\}$ indicates the type of non-terminal ICE, corresponding to ${E}_{1}$ (PD) and ${E}_{2}$ (TD). As there were almost no QoL data after TD, ${t}_{ij2}$ was kept constant at 0 after ${T}_{i2}$, assuming the same conditional effect of time-since-TD on QoL at, and at any time after TD. When a measurement was taken after the event, this was encoded by a time-varying indicator for TD. Analogously, for PD ${t}_{ij1}$ was kept constant from 3 cycles after ${T}_{i1}$(at -3). The baseline covariates age, sex and ECOG status were also recorded.

All time variables were modelled flexibly with splines to allow for possible nonlinear time-dependencies of QoL. We preferred restricted cubic splines (rcs) but opted for B-splines in some situations where the near-collinearity of rcs basis functions inhibited the convergence of the imputation model [47]. Discontinuation of treatment was observed in all study participants at some point, as participants only had access to the treatment within the study. As death and PD were not observed in all study participants, we included interactions between the time-to-ICE and the event indicator, so that a different relation was assumed between the PRO and the ICE itself versus the PRO and the censoring of the ICE.

#### Imputation models

For comparison, we applied several MI-FCS methods to our data. As there was only one variable with missing values in the data (QoL), only one conditional model needed to be specified (for QoL) and the process of iterating through several conditional models in each imputation was unnecessary. The number of imputations should be large enough to sufficiently approximate the predictive distribution of the missing values given the observed data (i.e., with sufficiently small Monte Carlo errors). While other suggestions have been made [48], a rule of thumb is that a number of imputations equal to the percentage of missing values in the data will suffice [49, 50]. In our case, 40.5% of QoL measurements were missing. Therefore, the number of imputations was set to 40 in our analyses. The MI-FCS models implemented are described in detail below (see also the R code provided in Online Resource 2).

First, we implemented multiple imputation with chained equations [41] (MICE), for multilevel data using two different approaches. For our first MICE model (model 1), we specified a linear multilevel model for QoL with heterogeneous within-patient variances and random effects on all predictors (‘2l.norm’ method in the mice package [41]). The model can be expressed in pseudo-code as: ${Y}_{ij}\sim (1 +\text{bspline}\left(j\right)+\text{bspline}\left({t}_{ij1}\right):{E}_{i1} + {E}_{i1} +\text{bspline}\left({t}_{ij2}\right)+\text{spline}\left({s}_{ij}\right): {D}_{i} + {D}_{i} + {E}_{ij1} + {E}_{ij2} + {\text{age}}_{\text{i}} + {\text{sex}}_{\text{i}} + {\text{ECOG}}_{\text{i}}\left|i\right.)$.

Our second MICE model (model 2) was a predictive mean matching algorithm based on a linear mixed model for QoL with a random patient intercept (the ‘2l.pmm’ method in the miceadds package [51]). In pseudo-code, the model is defined as: ${Y}_{ij}\sim \left(1|i\right) +\text{bspline}\left(j\right)+\text{bspline}\left({t}_{ij1}\right):{E}_{i1} + {E}_{i1} +\text{bspline}\left({t}_{ij2}\right)+\text{bspline}\left({s}_{ij}\right): {D}_{i} + {D}_{i} + {E}_{ij1}: {E}_{i1} + {E}_{ij2} + {\text{age}}_{\text{i}} + {\text{sex}}_{\text{i}} + {\text{ECOG}}_{\text{i}}$.

Both MICE approaches required the use of B-splines as the rcs basis vectors created issues with model convergence due to near-collinearity. The splines for the time-till-ICE variables all had four knots located at 1, 4, 9 and 20 cycles before the ICE occurred. As a default, the mice package requires the conditional imputation models to be specified through a predictor matrix that indicates which other variables in the data to use for imputation of each incomplete variable. To include interactions, splines or other nonlinear terms in the imputation, the user must add variables to the data that encode these terms. For splines, this entails manually computing spline bases and adding them as variable columns to the data. The ‘formulas’ argument in the ‘mice’ function may offer an easier way to specify nonlinear terms in an imputation model using standard R formula syntax. However, it is currently unclear how to define a cluster variable for multilevel imputation models in this way, and therefore we relied on model specification through the predictor matrix.

Second, we fitted a flexible linear mixed model to the data with a random patient-intercept using the lme4 package [52] (model 3). Again, the knots for the time-till-ICE splines were located at 1, 4, 9 and 20 cycles before occurrence of the ICE. We subsequently used the ‘predictInterval’ function from the package merTools [53] to draw imputations from (an approximation of) the conditional predictive distribution of the QoL variable. The model structure was as follows: ${Y}_{ij}\sim \left(1|i\right) +\text{rcs}\left(j\right)+\text{rcs}\left({t}_{ij1}\right)+ \text{rcs}\left({t}_{ij2}\right)+{\text{rcs}\left({s}_{ij}\right)}^{*}{D}_{i} + {E}_{ij1} + {E}_{ij1}: {E}_{i1} + {E}_{ij2} + {\text{age}}_{\text{i}} + {\text{sex}}_{\text{i}} + {\text{ECOG}}_{\text{i}}$. The interaction between time-till-PD (${t}_{ij1}$) and PD (${E}_{i1}$) was dropped because the model was over-parametrized. The advantages of this method compared to MICE were that we could specify the imputation model as a formula instead of a predictor matrix, and that we were able to incorporate restricted cubic splines. An obvious disadvantage is that this method is only applicable when QoL is the only incomplete variable.

Third, we used the ‘aregImpute’ function in the Hmisc package [42] to implement generalized additive regression models for multiple imputation. While these are not multilevel models, in the process of drawing imputations a clustered bootstrap is used to account for the multilevel structure of the data. Two different outcome models were specified: (a) a conditional normal model for QoL (model 4); and (b) conditional predictive mean matching for QoL (model 5). The model structure for both can be translated to pseudo-code as follows: ${Y}_{ij}\sim \text{rcs}\left(j\right)+{\text{rcs}\left({t}_{ij1}\right)}^{*}{E}_{i1}+ \text{rcs}\left({t}_{ij2}\right)+{\text{rcs}\left({s}_{ij}\right)}^{*}{D}_{i} + {E}_{ij1} + {E}_{ij1}: {E}_{i1} + {E}_{ij2} + \text{age}_{i}+\text{ sex}_{i} +\text{ ECOG}_{i}$.

All splines were restricted cubic splines with four knots at automatically selected locations.

All corresponding R code is available in Online Resource 2, which includes an example of how we coded the variables in our dataset.

#### Imputation results

For each of the methods, the QoL distribution in the imputed datasets was shifted somewhat to the left compared to the available data (Figure S2, Online Resource 1), suggesting that imputed QoL values were on average lower than the available values. A possible reason for this is that our imputation models detected a decrease in QoL in the weeks before death and around PD in the observed data, while patients were less likely to fill out questionnaires during these weeks, which was then accounted for in the imputation. For the mice 2l.pmm method, the distributions of QoL data in the respective imputed datasets differed more between imputations than for the other methods.

### Statistical analysis

After the imputation of missing data, the mean QoL while alive at each cycle was estimated using GEE with the cycle number as a categorical variable and an independence correlation structure [11, 30]. In the available data (model 0), this led to the same point estimates as simply calculating the sample mean QoL within the group of patients still alive at each respective cycle. We assumed non-informative censoring for overall survival (and other measurements) in our analyses, as most censoring was administrative at study end.

To compare our imputation methods to a reweighting approach (IPW-GEE, model 6), we also estimated the mean QoL while alive using the same GEE as above but on the incomplete data and weighted by the inverse probability of a QoL measurement being available. The weights were estimated using a flexible logistic regression model containing the same independent variables as the imputation models, and were subsequently stabilized and truncated.

Finally, to assess the result of not including information about ICEs in the imputation, we fitted the same GEE (without weights) to a dataset with QoL imputed based on a linear mixed model with only a random patient-intercept and forward-time (cycle number) as a spline, and the baseline covariates age, sex and ECOG status (i.e., a naive linear mixed model where ICE information is not included, model 7).

### Analysis results

**Fig. 2:**
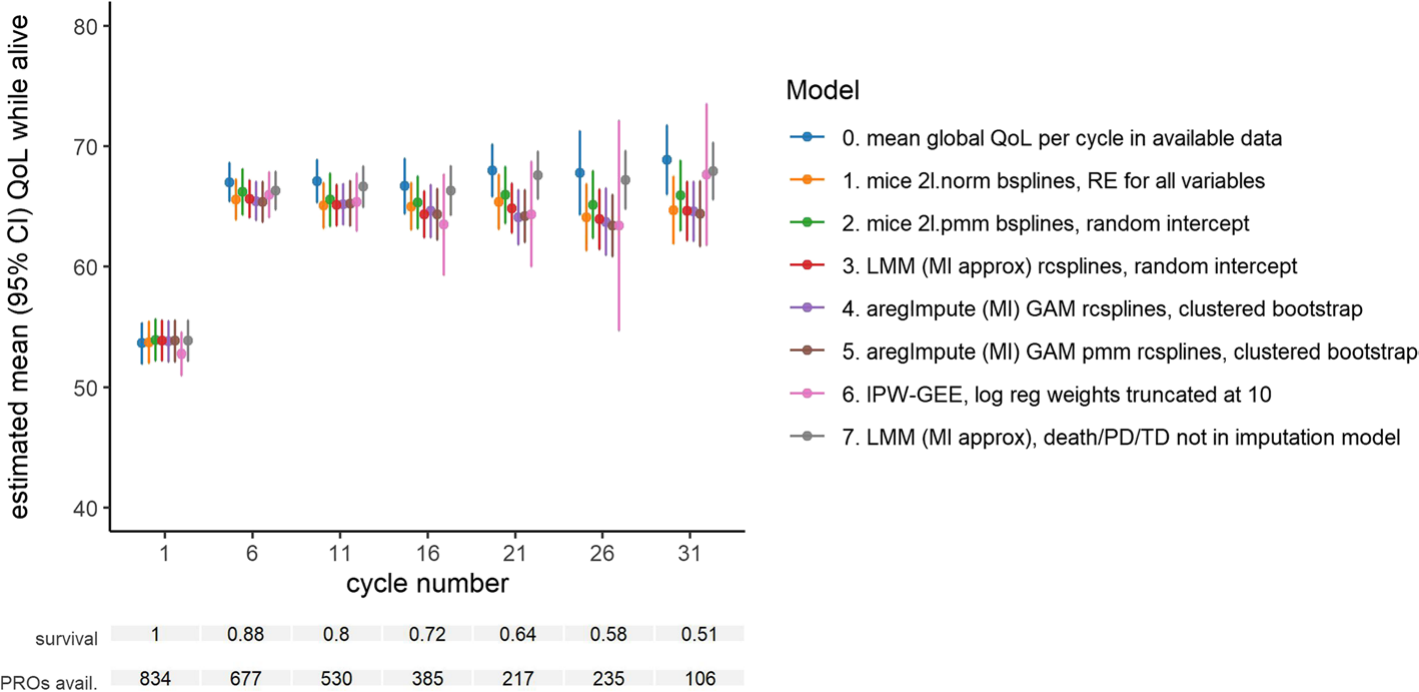
Estimated mean global QoL while alive in the available data (blue) and when using various methods to address missing data (other colors). Survival probabilities were estimated using the Kaplan–Meier method. *RE: random effects, LMM: linear mixed model, MI: multiple imputation, GAM: generalized additive model, rcsplines: restricted cubic splines, pmm: predictive mean matching, IPW-GEE: inverse probability weighted generalized estimating equations, log reg: logistic regression, PD: progression of disease, TD: treatment discontinuation

The imputation models 1–5 described in Sect. 3.3.2 resulted in very similar estimated mean QoL while alive over time (Fig. 2). Only the two-level predictive mean matching model (model 2) showed slightly higher estimates than the others. The results from the reweighting approach (model 6) closely follow the imputation results in the first 20 cycles but become unstable at later cycles when some patient types become rare and therefore some patients had much larger weights than others. Even in early cycles, there were some patients with larger weights than others (e.g., those who died early or experienced early PD), which led to mean QoL estimates that were slightly different from the imputation methods’ results. Importantly, all imputation methods that did account for ICEs (models 1–5) led to lower estimated mean QoL compared to the available data (model 0) and to the imputation without ICE information (model 7). The mean QoL resulting from models 1–5 was similar to the mean QoL in the available data at early cycles but the difference increased to about 5 points in mean QoL at later cycles when there were more missing values and more ICEs had occurred.

Regarding statistical efficiency, the standard errors resulting from most imputation methods were similar, providing no clear reason to prefer one over the others (Fig. 2). The two-level predictive mean matching model (model 2) did result in wider confidence intervals (CIs) than other methods. The IPW-GEE method (model 6) provided CIs similar to the imputation methods in the earlier cycles, but showed large uncertainty at later cycles, in line with the instability in the corresponding point estimates.

## Discussion

In this study, we have shown how imputation models for longitudinal PROs can be implemented that incorporate information about ICEs and that do not impute PROs after death. In our example, accounting for death and other ICEs in the imputation of missing HRQoL data led to lower estimated mean HRQoL over time compared to an available case analysis and a naive linear mixed model analysis. The information about the ICEs may (partly) capture that patients in a worse state are less likely to fill out questionnaires while they are more likely to have lower HRQoL values. In general, incorporating information about the timing and occurrence of ICEs when handling missing PRO data in a study is important as it makes a MAR assumption more plausible. In analyses that do not consider ICE information when handling missing PRO data, the extent of the bias will depend on the strength of the underlying relation between the ICEs and the missing values, the number of ICEs that occur and the total amount of missing data.

Our findings are in line with a recent study by Haug et al. [33], who implemented an IPW method accounting for the time to death and other auxiliary variables to handle missing HRQoL data in two cancer studies. Their results indicated that, at each time point, the mean HRQoL was likely lower in the group of participants who did not report HRQoL, compared to the group who did report HRQoL. Our study reiterates the importance of incorporating not only death, but all available information about ICEs in the missing data strategy in alignment with the estimand.

### Implementation

In our example, the IPW method showed instability at later cycles when some observations had a very high chance of being missing. Between the ICE-sensitive imputation methods in our example, the resulting estimates and standard errors were quite similar. There were, however, some differences in the computational time taken for the imputation: the aregImpute function was relatively fast, whereas the MICE 2l.norm method took longest. However, the MICE 2l.norm method implements a model with many random effects and heterogeneous within-patient variances, which the aregImpute function does not, so it makes sense that MICE takes more time. When the longitudinal PRO is the only incomplete variable in the data, fitting a flexible linear mixed model with ICE information and then drawing imputations from an approximation of the resulting predictive distribution is easy to implement and has a relatively low computational time.

All multiple imputation models implemented in our example were MI-FCS methods that target the joint posterior distribution of incomplete variables by specification of conditional univariate distributions. A formula-based method to specify interactions, splines and other nonlinear terms in multilevel imputation models would be a useful future addition to the ‘mice’ software. Such a method would be more user-friendly than model specification through a predictor matrix as shown in this paper. Multiple imputation by modelling the joint posterior distribution directly that takes the multilevel data structure into account has also been described in the literature [54, 55] (R software includes the jomo package [56]). We were, however, not able to arrive at a working implementation of joint modeling MI using this software with our example data. Further research might explore joint modeling MI methods for longitudinal PRO data.

### Generalizability of illustrated methods

While we illustrated our methods in data from a single arm trial, the general conclusion that ICE information is helpful in the imputation of missing PRO data holds for randomized studies as well. The methods shown for the single arm study can readily be applied to randomized trials, with the addition of treatment assignment to the predictor variables in the imputation. Furthermore, we showed results for a while-alive estimand in our examples, using GEE as the substantive analysis model after imputation. However, the same imputation methods shown can be combined with other substantive analysis models to arrive at different estimands of interest [11].

For example, suppose interest is in a composite estimand for death where an outcome is defined as the HRQoL value before death and 0 after. In that case, one can use the imputed HRQoL data for each patient up to death and add zeroes after death, until the end of the time horizon, after which the imputed outcomes are analyzed. If interest is in a hypothetical estimand for death, one may, for instance, estimate a mixed model on the imputed data until death. To estimate a hypothetical estimand for treatment discontinuation, a mixed model can be fitted only on the (imputed) data before treatment discontinuation. For a while-on-treatment estimand, one can analyze the (imputed) data before treatment discontinuation using GEE. Of course, in the latter case, the imputation could be limited to PRO data until treatment discontinuation. This can be done using the same methods as described above but with a dataset that has rows for each patient until treatment discontinuation, rather than until death.

This study focused on the imputation of PROs on their original numerical scale. Once missing values have been imputed, various analyses can be performed, such as estimation of the mean PRO at timepoints of interest (as in our examples) or a change from baseline in the PRO. Sometimes, numerical PRO values variables are dichotomized, for example, to classify participants into responders and non-responders. The disadvantages of a PRO (non-)responder analysis in terms of bias and reduction of statistical power have been discussed in the literature [57, 58]. If dichotomization of PRO values is still desired, we recommend imputing the PROs on the original numerical scale first and then dichotomizing them in each imputed dataset before performing the final analysis [59].

### Limitations

An important limitation of our trial data example is that there was very little available information in some regions of the data space, e.g., after TD. Therefore, model assumptions weigh heavily there (for example, setting time-till-TD to 0 after TD). This is emphasized by the instability of the IPW-GEE approach at later cycles. When data are not collected after TD, the estimation of a while-alive, treatment policy estimand relies strongly on untestable assumptions. Generally, at the study design phase, care should be taken that planned data collection methods align with the estimand of interest.

Furthermore, we can never be sure that the relation between the ICEs and the PRO in the observed data is the same in the missing data. In other words, the MAR assumption is untestable. Therefore, in a real trial analysis, we recommend performing sensitivity analyses to assess the impact of imputation model assumptions on the resulting estimates. If PRO data are intended for review of payer or regulatory submission, discussions with these stakeholders to identify methods to handle missing data that align with appropriate estimands are highly encouraged.

## Conclusion

To account for ICE information when handling missing PRO data, the missing data model should be separated from the analysis model as ICEs may occur later than the missed measurement. Therefore, MI and IPW methods are preferred over direct likelihood-based methods to handle missing data. The choice between MI and IPW depends on whether one believes to have an appropriate model for the missing outcomes themselves or for the probability of missingness, respectively. In an example dataset, we have illustrated how information about intercurrent events can be incorporated in the multiple imputation of a continuous, longitudinal PRO. Further research is required to formally compare imputation methods that include ICE information. Whichever method is applied, it is important that information about the timing and occurrence of ICEs is taken into account when handling missing PRO data.

## Supplementary Information

Supplementary Material 1.

Supplementary Material 2.

## Data Availability

The data that support the findings of this study are available from Pfizer, Inc., but restrictions apply to the availability of these data, which were used under license for the current study, and so are not publicly available. Data are however available from the authors upon reasonable request and with permission of Pfizer, Inc.

## References

[1] European Medicines Agency. Appendix 2 to the guideline on the evaluation of anticancer medicinal products in man. The use of patient-reported outcome (PRO) measures in oncology studies. European Medicines Agency; 2016. Available from: https://www.ema.europa.eu/en/documents/other/appendix-2-guideline-evaluation-anticancer-medicinal-products-man_en.pdf. Cited 2023 Apr 28.

[2] U.S. Department of Health and Human Services, Food and Drug Administration, Center for Drug Evaluation and Research (CDER), Center for Biologics Evaluation and Research (CBER). Patient-Focused Drug Development: Methods to Identify What Is Important to Patients - Guidance for Industry, Food and Drug Administration Staff, and Other Stakeholders (Final Guidance). 2022. Available from: https://www.fda.gov/media/131230/download. Cited 2023 Oct 18.

[3] U.S. Department of Health and Human Services Food and Drug Administration. Guidance for Industry - Submitting Patient-Reported Outcome Data in Cancer Clinical Trials. 2023. Available from: https://www.fda.gov/media/173581/download. Cited 2024 Mar 18.

[4] U.S. Department of Health and Human Services Food and Drug Administration. Guidance for Industry - Core Patient-Reported Outcomes in Cancer Clinical Trials (Draft Guidance). 2021. Available from: https://www.fda.gov/media/149994/download. Cited 2023 Aug 16.

[5] Liu L, Choi J, Musoro JZ, Sauerbrei W, Amdal CD, Alanya A, et al. Single-arm studies involving patient-reported outcome data in oncology: a literature review on current practice. Lancet Oncol. 2023;24:e197–206. 37142381 10.1016/S1470-2045(23)00110-9

[6] Teixeira MM, Borges FC, Ferreira PS, Rocha J, Sepodes B, Torre C. A review of patient-reported outcomes used for regulatory approval of oncology medicinal products in the European Union between 2017 and 2020. Front Med. 2022;9:968272. 10.3389/fmed.2022.968272 PMC9411861 36035431

[7] Pe M, Dorme L, Coens C, Basch E, Calvert M, Campbell A, et al. Statistical analysis of patient-reported outcome data in randomised controlled trials of locally advanced and metastatic breast cancer: a systematic review. Lancet Oncol. 2018;19:e459–69. 30191850 10.1016/S1470-2045(18)30418-2

[8] Fiero MH, Roydhouse JK, Vallejo J, King-Kallimanis BL, Kluetz PG, Sridhara R. US Food and Drug Administration review of statistical analysis of patient-reported outcomes in lung cancer clinical trials approved between January, 2008, and December, 2017. Lancet Oncol. 2019;20:e582–9. 31579004 10.1016/S1470-2045(19)30335-3

[9] European Medicines Agency. ICH E9 (R1) addendum on estimands and sensitivity analysis in clinical trials to the guideline on statistical principles for clinical trials. European Medicines Agency; 2020. Available from: https://www.ema.europa.eu/en/documents/scientific-guideline/ich-e9-r1-addendum-estimands-sensitivity-analysis-clinical-trials-guideline-statistical-principles_en.pdf. Cited 2023 Apr 28.

[10] Fiero MH, Pe M, Weinstock C, King-Kallimanis BL, Komo S, Klepin HD, et al. Demystifying the estimand framework: a case study using patient-reported outcomes in oncology. Lancet Oncol. 2020;21:e488–94. 33002444 10.1016/S1470-2045(20)30319-3

[11] Thomassen D, Roychoudhury S, Amdal CD, Reynders D, Musoro JZ, Sauerbrei W, et al. The role of the estimand framework in the analysis of patient-reported outcomes in single-arm trials: a case study in oncology. BMC Med Res Methodol. 2024;24:290. 39580440 10.1186/s12874-024-02408-x PMC11585159

[12] Wolbers M, Noci A, Delmar P, Gower-Page C, Yiu S, Bartlett JW. Standard and reference-based conditional mean imputation. Pharm Stat. 2022;21:1246–57. 35587109 10.1002/pst.2234 PMC9790242

[13] Noci A, Wolbers M, Abt M, Baayen C, Burger HU, Jin M, et al. A Comparison of estimand and estimation strategies for clinical trials in early Parkinson’s disease. Stat Biopharm Res. 2023;15:491–501.

[14] Bell J, Drury T, Mütze T, Pipper CB, Guizzaro L, Mitroiu M, et al. Estimation methods for estimands using the treatment policy strategy; a simulation study based on the PIONEER 1 Trial. arXiv; 2024. Available from: http://arxiv.org/abs/2402.12850. Cited 2024 Apr 29. 10.1002/pst.2472 40045733

[15] Mallinckrodt CH, Bell J, Liu G, Ratitch B, O’Kelly M, Lipkovich I, et al. Aligning estimators with estimands in clinical trials: putting the ICH E9(R1) guidelines into practice. Ther Innov Regul Sci. 2020;54:353–64. 32072593 10.1007/s43441-019-00063-9

[16] Carpenter JR, Roger JH, Kenward MG. Analysis of longitudinal trials with protocol deviation: a framework for relevant, accessible assumptions, and inference via multiple imputation. J Biopharm Stat. 2013;23:1352–71. 24138436 10.1080/10543406.2013.834911

[17] Detry MA, Ma Y. Analyzing repeated measurements using mixed models. JAMA. 2016;315:407. 26813213 10.1001/jama.2015.19394

[18] Mallinckrodt CH, Lane PW, Schnell D, Peng Y, Mancuso JP. Recommendations for the primary analysis of continuous endpoints in longitudinal clinical trials. Drug Inf J. 2008;42:303–19.

[19] Bell ML, Floden L, Rabe BA, Hudgens S, Dhillon HM, Bray VJ, et al. Analytical approaches and estimands to take account of missing patient-reported data in longitudinal studies. Patient Relat Outcome Meas. 2019;10:129–40. 31114411 10.2147/PROM.S178963 PMC6489631

[20] Cella D, Li JZ, Cappelleri JC, Bushmakin A, Charbonneau C, Kim ST, et al. Quality of life in patients with metastatic renal cell carcinoma treated with sunitinib or interferon alfa: results from a phase iii randomized trial. J Clin Oncol. 2008;26:3763–9. 18669464 10.1200/JCO.2007.13.5145

[21] Chase DM, Marín MR, Backes F, Han S, Graybill W, Mirza MR, et al. Impact of disease progression on health-related quality of life of advanced ovarian cancer patients – Pooled analysis from the PRIMA trial. Gynecol Oncol. 2022;166:494–502. 35851489 10.1016/j.ygyno.2022.06.028

[22] Marschner N, Zacharias S, Lordick F, Hegewisch-Becker S, Martens U, Welt A, et al. Association of disease progression with health-related quality of life among adults with breast, lung, pancreatic, and colorectal cancer. JAMA Netw Open. 2020;3:e200643. 32154886 10.1001/jamanetworkopen.2020.0643 PMC7064873

[23] Elmqvist MA, Jordhøy MS, Bjordal K, Kaasa S, Jannert M. Health-related quality of life during the last three months of life in patients with advanced cancer. Support Care Cancer. 2009;17:191–8. 18581147 10.1007/s00520-008-0477-2

[24] Diehr P, Lafferty WE, Patrick DL, Downey L, Devlin SM, Standish LJ. Quality of life at the end of life. Health Qual Life Outcomes. 2007;5:51. 17683554 10.1186/1477-7525-5-51 PMC2077331

[25] Li Z, Tosteson TD, Bakitas MA. Joint modeling quality of life and survival using a terminal decline model in palliative care studies. Stat Med. 2013;32:1394–406. 23001893 10.1002/sim.5635 PMC3623280

[26] Claessens AKM, Ramaekers BLT, Lobbezoo DJA, Van Kampen RJW, De Boer M, Van De Wouw AJ, et al. Quality of life in a real-world cohort of advanced breast cancer patients: a study of the SONABRE Registry. Qual Life Res. 2020;29:3363–74. 32816222 10.1007/s11136-020-02604-4 PMC7686224

[27] Verkissen MN, Hjermstad MJ, Van Belle S, Kaasa S, Deliens L, Pardon K. Quality of life and symptom intensity over time in people with cancer receiving palliative care: Results from the international European Palliative Care Cancer Symptom study. Montazeri A, editor. PLOS ONE. 2019;14:e0222988. 31596849 10.1371/journal.pone.0222988 PMC6784977

[28] Giesinger JM, Wintner LM, Oberguggenberger AS, Gamper EM, Fiegl M, Denz H, et al. Quality of life trajectory in patients with advanced cancer during the last year of life. J Palliat Med. 2011;14:904–12. 21711125 10.1089/jpm.2011.0086

[29] Pe M, Alanya A, Falk RS, Amdal CD, Bjordal K, Chang J, et al. Setting International Standards in Analyzing Patient-Reported Outcomes and Quality of Life Endpoints in Cancer Clinical Trials-Innovative Medicines Initiative (SISAQOL-IMI): stakeholder views, objectives, and procedures. Lancet Oncol. 2023;24:e270–83. 37269858 10.1016/S1470-2045(23)00157-2

[30] Kurland BF, Heagerty PJ. Directly parameterized regression conditioning on being alive: analysis of longitudinal data truncated by deaths. Biostatistics. 2005;6:241–58. 15772103 10.1093/biostatistics/kxi006

[31] Olarte Parra C, Daniel RM, Bartlett JW. Hypothetical estimands in clinical trials: a unification of causal inference and missing data methods. Stat Biopharm Res. 2023;15:421–32. 37260584 10.1080/19466315.2022.2081599 PMC10228513

[32] Shardell M, Miller RR. Weighted estimating equations for longitudinal studies with death and non-monotone missing time-dependent covariates and outcomes. Stat Med. 2008;27:1008–25. 17579923 10.1002/sim.2964 PMC2792882

[33] Haug N, Jänicke M, Kasenda B, Marschner N, Frank M. Quantifying bias due to missing data in quality of life surveys of advanced-stage cancer patients. Qual Life Res. 2024;33:1085–94. 38240915 10.1007/s11136-023-03588-7

[34] Seaman SR, White IR. Review of inverse probability weighting for dealing with missing data. Stat Methods Med Res. 2013;22:278–95. 21220355 10.1177/0962280210395740

[35] Vansteelandt S, Carpenter J, Kenward MG. Analysis of incomplete data using inverse probability weighting and doubly robust estimators. Methodology. 2010;6:37–48.

[36] Tilling K, Williamson EJ, Spratt M, Sterne JAC, Carpenter JR. Appropriate inclusion of interactions was needed to avoid bias in multiple imputation. J Clin Epidemiol. 2016;80:107–15. 27445178 10.1016/j.jclinepi.2016.07.004 PMC5176003

[37] Engels JM, Diehr P. Imputation of missing longitudinal data: a comparison of methods. J Clin Epidemiol. 2003;56:968–76. 14568628 10.1016/s0895-4356(03)00170-7

[38] Huque MH, Carlin JB, Simpson JA, Lee KJ. A comparison of multiple imputation methods for missing data in longitudinal studies. BMC Med Res Methodol. 2018;18:168. 30541455 10.1186/s12874-018-0615-6 PMC6292063

[39] R Core Team. R: A language and environment for statistical computing. Vienna, Austria: R Foundation for Statistical Computing; 2022. Available from: https://www.R-project.org/.

[40] Van Buuren S. Multiple imputation of discrete and continuous data by fully conditional specification. Stat Methods Med Res. 2007;16:219–42. 17621469 10.1177/0962280206074463

[41] Buuren SV, Groothuis-Oudshoorn K. mice: Multivariate Imputation by Chained Equations in R. J Stat Softw. 2011;45. Available from: http://www.jstatsoft.org/v45/i03/. Cited 2024 May 17.

[42] Harrell FE. Hmisc: Harrell Miscellaneous. 2022. Available from: https://CRAN.R-project.org/package=Hmisc.

[43] Rouanet A, Helmer C, Dartigues J-F, Jacqmin-Gadda H. Interpretation of mixed models and marginal models with cohort attrition due to death and drop-out. Stat Methods Med Res. 2019;28:343–56. 28784010 10.1177/0962280217723675

[44] Blackhall F, Ross Camidge D, Shaw AT, Soria J-C, Solomon BJ, Mok T, et al. Final results of the large-scale multinational trial PROFILE 1005: efficacy and safety of crizotinib in previously treated patients with advanced/metastatic ALK-positive non-small-cell lung cancer. ESMO Open. 2017;2:e000219. 29209525 10.1136/esmoopen-2017-000219 PMC5703388

[45] Fayers P, Aaronson NK, Bjordal K, Groenvold M, Curran D, Bottomley A. EORTC QLQ-C30 Scoring Manual. Third edition. Brussels, Belgium: European Organisation for Research and Treatment of Cancer; 2001.

[46] Fayers P, Bottomley A. Quality of life research within the EORTC—the EORTC QLQ-C30. Eur J Cancer. 2002;38:125–33. 10.1016/s0959-8049(01)00448-8 11858978

[47] Perperoglou A, Sauerbrei W, Abrahamowicz M, Schmid M. A review of spline function procedures in R. BMC Med Res Methodol. 2019;19:46. 30841848 10.1186/s12874-019-0666-3 PMC6402144

[48] Von Hippel PT. How many imputations do you need? A two-stage calculation using a quadratic rule. Sociol Methods Res. 2020;49:699–718. 39211325 10.1177/0049124117747303 PMC11361408

[49] Bodner TE. What improves with increased missing data imputations? Struct Equ Model Multidiscip J. 2008;15:651–75.

[50] White IR, Royston P, Wood AM. Multiple imputation using chained equations: issues and guidance for practice. Stat Med. 2011;30:377–99. 21225900 10.1002/sim.4067

[51] Robitzsch A, Grund S. miceadds: Some Additional Multiple Imputation Functions, Especially for “mice”. 2024. Available from: https://CRAN.R-project.org/package=miceadds.

[52] Bates D, Mächler M, Bolker B, Walker S. Fitting Linear Mixed-Effects Models Using lme4. J Stat Softw. 2015;67. Available from: http://www.jstatsoft.org/v67/i01/. Cited 2024 Jun 11.

[53] Jared E. Knowles, Carl Frederick, Alex Whitworth. merTools: Tools for Analyzing Mixed Effect Regression Models. 2024. Available from: https://cran.r-project.org/web/packages/merTools/merTools.pdf.

[54] Carpenter JR, Kenward MG. Multiple Imputation and its Application. 1st ed. Wiley; 2013. Available from: https://onlinelibrary.wiley.com/doi/book/10.1002/9781119942283. Cited 2024 May 17.

[55] Quartagno M, Carpenter JR. Multiple imputation for IPD meta-analysis: allowing for heterogeneity and studies with missing covariates. Stat Med. 2016;35:2938–54. 26681666 10.1002/sim.6837 PMC5064632

[56] Quartagno M, Grund S, Carpenter J. jomo: a flexible package for two-level joint modelling multiple imputation. R J. 2019;11:205.

[57] Cappelleri JC, Chambers R. Addressing bias in responder analysis of patient-reported outcomes. Ther Innov Regul Sci. 2021;55:989–1000. 34046875 10.1007/s43441-021-00298-5 PMC8332587

[58] Cappelleri JC. Further reduction in statistical power for responder analysis of patient-reported outcomes with measurement error. J Clin Epidemiol. 2021;140:200–1. 34416325 10.1016/j.jclinepi.2021.08.017

[59] Floden L, Bell ML. Imputation strategies when a continuous outcome is to be dichotomized for responder analysis: a simulation study. BMC Med Res Methodol. 2019;19:161. 31345166 10.1186/s12874-019-0793-x PMC6659229

