# Supplementary material for: Handling missing values in patient-reported outcome data in the presence of intercurrent events
Source: BMC Med Res Methodol. 2025 Mar 1;25:56. doi: 10.1186/s12874-025-02510-8 (PMC11872335; doi:10.1186/s12874-025-02510-8)
Supplement: Supplementary file 1 — Supplementary Material 1. [file 12874_2025_2510_MOESM1_ESM.pdf]

Online Resource 1: Supplementary Figures

Article title “Handling missing values in patient-reported outcome data in the presence of intercurrent events”

Authors Doranne Thomassen[1], Satrajit Roychoudhury[2], Cecilie Delphin Amdal[3,4], Dries Reynders[5], Jammbe Z. Musoro[6], Willi Sauerbrei[7], Els Goetghebeur[5], Saskia le Cessie[1,5,8]*; on behalf of SISAQOL-IMI Work Package 3**

[1]Department of Biomedical Data Sciences, Leiden University Medical Center, Leiden, The Netherlands.
[2] Pfizer Inc, New York, NY, USA.
[3] Research Support Services, Oslo University Hospital, Oslo, Norway.
[4] Department of Oncology, Oslo University Hospital, Oslo, Norway.
[5] Department of Applied Mathematics, Computer Science and Statistics, Ghent University, Ghent, Belgium.
[6] European Organisation for Research and Treatment of Cancer (EORTC) Headquarters, Brussels, Belgium.
[7] Institute of Medical Biometry and Statistics, Faculty of Medicine and Medical Center - University of Freiburg, Freiburg, Germany.
[8] Department of Clinical Epidemiology, Leiden University Medical Center, Leiden, The Netherlands.
* Corresponding author. Email address for correspondence: s.le_cessie@lumc.nl.
** A list of authors and their affiliations appears at the end of the paper.

Timepoint (j)	1	2	3	4	5	6	7	8	9	10	11	12	13	14
PRO measurement Y_{ij}<br>O=available<br>X=missing	**O**	**O**	**O**	**O**	**X**	**O**	**X**	**X**	**O**	**X**	**X**	**X**	**-**	**-**
Intercurrent events<br>E_{ik}, S_i								PD<br>$E_{i1} = 1$<br>$T_{i1} = 8$	TD<br>$E_{i2} = 1$<br>$T_{i2} = 9$			Death<br>$D_i = 1$<br>$S_i = 12$		
E_{ij1}	0	0	0	0	0	0	0	1	1	1	1	1		
E_{ij2}	0	0	0	0	0	0	0	0	1	1	1	1		
Time till PD (t_{ij1})	7	6	5	4	3	2	1	0	-1	-2	-3	-4		
Time till TD (t_{ij2})	8	7	6	5	4	3	2	1	0	-1	-2	-3		
Time till death (s_{ij})	11	10	9	8	7	6	5	4	3	2	1	0		
X_i, e.g., patient age at baseline	67	67	67	67	67	67	67	67	67	67	67	67		

*Figure S1. Schematic representation of PRO data availability for a single example patient i throughout a study with 14 PRO measurement times. Corresponding variables that may be used for imputation of missing PRO data for patient i are shown below each planned measurement. *PD: progression of disease, TD: treatment discontinuation.*

For example, to impute the missing PRO value at timepoint $j = 5$, we use, besides the observed PRO information of patient i over time, that the patient did not experience disease progression yet, that the remaining time until observed disease progression is 3 timepoints, that discontinuation has not yet occurred but is observed 4 timepoints later, and that death was observed 7 timepoints later. Additionally, we can use other variables that are assumed to be informative for the missing PRO value such as the patient's age at baseline.

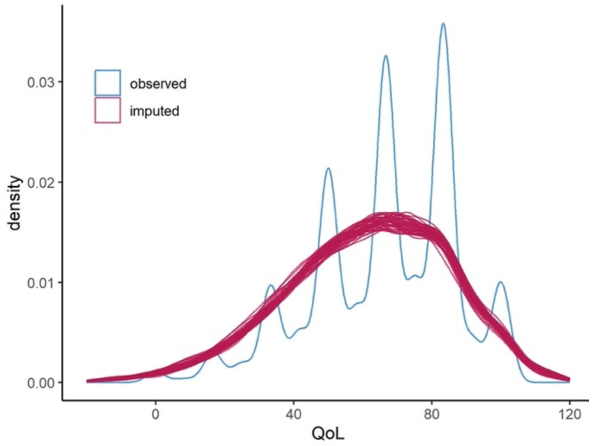

a. mice two-level normal model

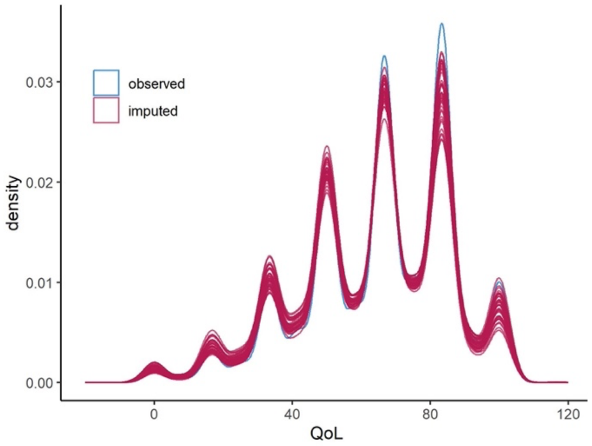

b. miceadds two-level pmm

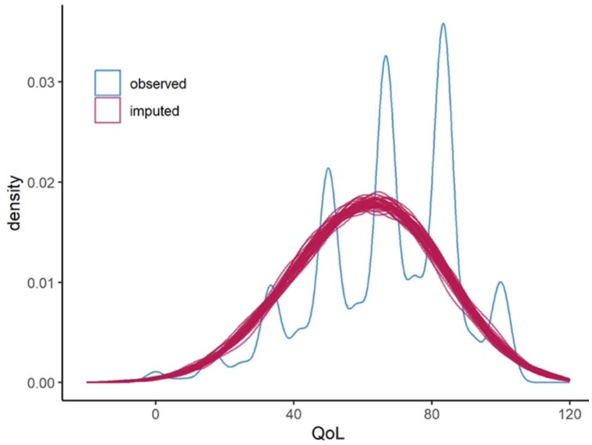

c. lme4 two-level normal model + predictInterval

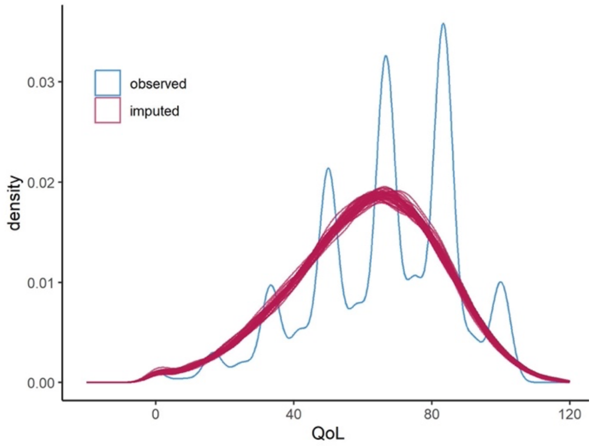

d. aregImpute normal model

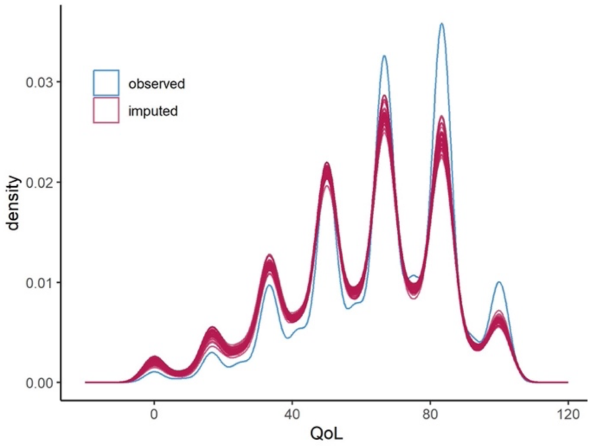

e. aregImpute predictive mean matching

Figure S2. The distribution of QoL data in the available data (blue) and the imputed datasets (red) for five implemented imputation methods.
