# Supplementary material for: Handling missing values in patient-reported outcome data in the presence of intercurrent events
Source: BMC Med Res Methodol. 2025 Mar 1;25:56. doi: 10.1186/s12874-025-02510-8 (PMC11872335; doi:10.1186/s12874-025-02510-8)
Supplement: Supplementary file 2 — Supplementary Material 2. [file 12874_2025_2510_MOESM2_ESM.html]

Handling missing values in patient-reported outcomes in the presence of intercurrent events – Online Supplement 2: Analysis Code


# Handling missing values in patient-reported outcomes in the presence of intercurrent events – Online Supplement 2: Analysis Code

#### Doranne Thomassen

#### 2024-09-27

- Article
  information
- Packages
  used
- Preparing the data for
  imputation
- Imputing
  missing data with various multiple imputation methods
  - Multiple imputation
    using aregImpute GAMs
  - Multiple
    imputation using chained equations: MICE
  - Multiple
    imputation from a linear mixed model
- Alternative:
  GEE weighted by the inverse probability of completeness
- Visualizing the results

# Article information

Article title: Handling missing values in patient-reported outcome
data in the presence of intercurrent events  
Authors: Doranne Thomassen[1], Satrajit Roychoudhury[2], Cecilie Delphin
Amdal[3,4], Dries Reynders[5], Jammbe Z. Musoro[6], Willi Sauerbrei[7],
Els Goetghebeur[5], Saskia le Cessie[1,5,8]\*; on behalf of SISAQOL-IMI
Work Package 3\*\*

1 Department of Biomedical Data Sciences, Leiden University Medical
Center, Leiden, The Netherlands.  
2 Pfizer Inc, New York, NY, USA.  
3 Research Support Services, Oslo University Hospital, Oslo,
Norway.  
4 Department of Oncology, Oslo University Hospital, Oslo, Norway.  
5 Department of Applied Mathematics, Computer Science and Statistics,
Ghent University, Ghent, Belgium.  
6 European Organisation for Research and Treatment of Cancer (EORTC)
Headquarters, Brussels, Belgium.  
7 Institute of Medical Biometry and Statistics, Faculty of Medicine and
Medical Center - University of Freiburg, Freiburg, Germany.  
8 Department of Clinical Epidemiology, Leiden University Medical Center,
Leiden, The Netherlands.  
\* Corresponding author. Email address for correspondence: s.le\_cessie@lumc.nl.  
\*\* A list of contributors and their affiliations appears at the end of
the paper.

# Packages used

```
require(Hmisc)
require(mice)
require(miceadds)
require(splines)
require(dplyr)
require(geepack)
require(glmtoolbox)
require(ggplot2)
require(ggmice)
require(lme4)
require(merTools)
require(arm)
```

# Preparing the data for imputation

We assume here that the data are already loaded. ‘QoL\_grid’ is a
longitudinal dataset with QoL measurements in long format, i.e., each
row contains measured QoL at one cycle (and possibly other
measurements/data at that cycle) and a patient identifier ‘patid’
indicating to which patient the measurement belongs. Each patient will
have several corresponding rows, one for each cycle number where QoL was
planned to be measured.

TD = treatment discontinuation; PD = disease progression; ECOGB =
ECOG status at baseline.

```
#Create factors from num/char vars
QoL_grid$patid <- as.factor(QoL_grid$patid)
QoL_grid$sex <- as.factor(QoL_grid$sex)
QoL_grid$SMOKING <- as.factor(QoL_grid$SMOKING)
QoL_grid$ECOGB <- as.factor(QoL_grid$ECOGB)

summary(QoL_grid)
```

```
# Average QoL in the available data over the cycles for comparison (model 0). This is all while alive and not censored for death.
library(plotrix)
obsmeans_grid <- aggregate(QoL~cycleno, data=QoL_grid, FUN = function(x){mean(x, na.rm=TRUE)})
obsmeans_SE_grid <- aggregate(QoL~cycleno, data=QoL_grid, FUN = function(x){std.error(x, na.rm=TRUE)})
```

```
##Create a dataframe in long format for imputation
#remove everything after cycle 40
maxcycles <- 40
QoL_grid <- QoL_grid[-which(QoL_grid$cycleno>maxcycles),]

#Remove all rows after the corresponding patient's death/censoring for OS
QoL_grid <- QoL_grid[-which(QoL_grid$cycleno>QoL_grid$lastcycle_no),]

#------------- coding the intercurrent events -------------------------------

# TD ----- Add time distance to Treatment Discontinuation with a minimum of 0
#TD is observed in everyone, since one can only be on the treatment while in the study
QoL_grid$cycles_till_TD <- as.numeric(QoL_grid$cycle_eoTx-QoL_grid$cycleno)
QoL_grid$cycles_till_TD[which(QoL_grid$cycles_till_TD < 0)] <- 0
QoL_grid$TD_yet <- as.numeric(QoL_grid$cycles_till_TD <=0)


# PD -----Add indicator of disease progession
# We assume all death is disease related in this study
QoL_grid$PD <- as.numeric(!is.na(QoL_grid$cycle_PD) | QoL_grid$died==1)
summary(QoL_grid$PD)

#Add time distance to PD with a mininum of -3
QoL_grid$cycles_till_PD <- as.numeric(QoL_grid$cycle_PD-QoL_grid$cycleno)
QoL_grid$cycles_till_PD[which(is.na(QoL_grid$cycles_till_PD) & QoL_grid$died==1)] <- QoL_grid$cycles_till_end[which(is.na(QoL_grid$cycles_till_PD) & QoL_grid$died==1)]

summary(QoL_grid$QoL[which(QoL_grid$cycles_till_PD < 0)])
summary(QoL_grid$cycles_till_PD[which(QoL_grid$cycles_till_PD < 0)])
length(QoL_grid$QoL[which(QoL_grid$cycles_till_PD < 0)])

QoL_grid$cycles_till_PD[which(QoL_grid$cycles_till_PD < -3)] <- -3

#  and which is censored at the time of TD if not observed
QoL_grid$cycles_till_PD[which(is.na(QoL_grid$cycles_till_PD))] <- QoL_grid$cycles_till_TD[which(is.na(QoL_grid$cycles_till_PD))]


#Time-varying indicator of whether PD has occurred yet in those with PD 
QoL_grid$PD_yet <- as.numeric(QoL_grid$cycles_till_PD <=0)


#---------------------------------------------------------------------------
QoL_grid$cycleno_factor <- as.factor(QoL_grid$cycleno)


QoL_grid$diedxtime <- QoL_grid$died * QoL_grid$cycles_till_end
QoL_grid$PDxtime <- QoL_grid$PD * QoL_grid$cycles_till_PD
QoL_grid$PD_yetxPD <- QoL_grid$PD * QoL_grid$PD_yet

## create bspline bases voor the "cycles_till" and cycle number variables for MICE

cyc_death_sp <- bs(x=QoL_grid$cycles_till_end, knots = c(1,4, 9,20))
colnames(cyc_death_sp) <- paste0("cyc_death_sp.", 1:ncol(cyc_death_sp))
N_spline_columns <- ncol(cyc_death_sp)
QoL_grid <- cbind(QoL_grid, cyc_death_sp)

## also add interaction terms with indicator variables
sp_interactions <- cyc_death_sp*QoL_grid$died
colnames(sp_interactions) <- paste0("diedxdeath.sp.", 1:ncol(sp_interactions))
QoL_grid <- cbind(QoL_grid, sp_interactions)
rm(cyc_death_sp)
rm(sp_interactions)

cyc_PD_sp <- bs(x=QoL_grid$cycles_till_PD, knots = c(1,4,9, 20))
colnames(cyc_PD_sp) <- paste0("cyc_PD_sp.", 1:ncol(cyc_PD_sp))
QoL_grid <- cbind(QoL_grid, cyc_PD_sp)
sp_interactions <- cyc_PD_sp*QoL_grid$PD
colnames(sp_interactions) <- paste0("PDxPD.sp.", 1:ncol(sp_interactions))
QoL_grid <- cbind(QoL_grid, sp_interactions)
rm(sp_interactions)
rm(cyc_PD_sp)

cyc_TD_sp <- bs(x=QoL_grid$cycles_till_TD, knots = c(1,4, 9,20))
colnames(cyc_TD_sp) <- paste0("cyc_TD_sp.", 1:ncol(cyc_TD_sp))
QoL_grid <- cbind(QoL_grid, cyc_TD_sp)
rm(cyc_TD_sp)

cycleno_sp <- bs(x=QoL_grid$cycleno, df=7)
colnames(cycleno_sp) <- paste0("cycleno_sp.", 1:ncol(cycleno_sp))
QoL_grid <- cbind(QoL_grid, cycleno_sp)
rm(cycleno_sp)

QoL_grid$patid_num <- as.integer(QoL_grid$patid)
```

# Imputing missing data with various multiple imputation methods

```
# Set the number of imputations 
n.imp <- 40
```

## Multiple imputation using aregImpute GAMs

```
#------------------------------------------------------------------
# Predictive mean matching imputation

imp_aReg <- aregImpute(QoL ~ cycleno + cycles_till_PD*PD + cycles_till_TD + cycles_till_end*died + PD_yet + PD_yetxPD + TD_yet + Age + sex + ECOGB, 
                       data=QoL_grid, 
                       group = QoL_grid$patid, 
                       nk=4,
                       n.impute = n.imp,
                       type = "pmm")

completed_long <- QoL_grid
completed_long$.imp <- 0
#completed_long$.id <- completed_long$patid

for(i in 1:n.imp){
  completed <- QoL_grid
  imputed <- impute.transcan(imp_aReg, imputation=i, data=QoL_grid, list.out=TRUE,
                            pr=FALSE, check=FALSE)
  completed[names(imputed)] <- imputed
  completed$.imp <- i
  #completed$.id <- completed$patid
  completed_long <- rbind(completed_long, completed)
}

#Analysis of mean QoL while alive using a GEE with independence correlation structure
aReg.mids_pmm <- as.mids(completed_long)
aReg.mids_pmm_pooled <- summary(pool(with(aReg.mids_pmm, geeglm(QoL~as.factor(cycleno)-1, id=patid ,corstr = "independence"))))

# Inspect the density of imputed vs available values
densityplot(aReg.mids_pmm, ~QoL)

ggmice(aReg.mids_pmm, aes(x = QoL, group = .imp)) +
  geom_density()+
  xlab("QoL")+
  xlim(c(-20, 120))+
  theme(legend.position = c(0.25, 0.8))
ggsave(filename = "imp_density_aregImp_pmm.jpg", height = 4, width = 5.33)

# Regression imputation
imp_aReg <- aregImpute(QoL ~ cycleno + cycles_till_PD*PD + cycles_till_TD + cycles_till_end*died + PD_yet + PD_yetxPD + TD_yet + Age + sex + ECOGB, 
                       data=QoL_grid, 
                       group = QoL_grid$patid, 
                       nk=4,
                       n.impute = n.imp,
                       type = "regression")

completed_long <- QoL_grid
completed_long$.imp <- 0
#completed_long$.id <- completed_long$patid

for(i in 1:n.imp){
  completed <- QoL_grid
  imputed <- impute.transcan(imp_aReg, imputation=i, data=QoL_grid, list.out=TRUE,
                            pr=FALSE, check=FALSE)
  completed[names(imputed)] <- imputed
  completed$.imp <- i
  #completed$.id <- completed$patid
  completed_long <- rbind(completed_long, completed)
}

#Analysis of mean QoL while alive using a GEE with independence correlation structure
aReg.mids_norm <- as.mids(completed_long)
aReg.mids_norm_pooled <- summary(pool(with(aReg.mids_norm, geeglm(QoL~as.factor(cycleno)-1, id=patid ,corstr = "independence"))))

# Inspect the density of imputed vs available values
densityplot(aReg.mids_norm, ~QoL, xlab="QoL")

ggmice(aReg.mids_norm, aes(x = QoL, group = .imp)) +
  geom_density()+
  xlab("QoL")+
  xlim(c(-20, 120))+
  theme(legend.position = c(0.25, 0.8))
ggsave(filename = "imp_density_aregImp_norm.jpg", height = 4, width = 5.33)
```

## Multiple imputation using chained equations: MICE

```
#---------------MICE 2l.norm method --------------------------
# 1. Initialization and setting the predictor matrix
init <-  mice(QoL_grid, maxit = 0)
cor_mat <- cor(select_if(QoL_grid, is.numeric))

pred <- init$predictorMatrix
pred[]<- 0
pred["QoL","patid_num"]<- -2
pred["QoL",c(paste0("cycleno_sp.", 1:N_spline_columns),
  
             "died", 
             #paste0("cyc_death_sp.", 1:N_spline_columns),
             paste0("diedxdeath.sp.", 1:N_spline_columns),

             paste0("cyc_TD_sp.", 1:N_spline_columns),
             "TD_yet",
             
             "PD", 
             "PD_yet",
             #"PD_yetxPD",
             #paste0("cyc_PD_sp.", 1:N_spline_columns),
             paste0("PDxPD.sp.", 1:N_spline_columns),
             
             "Age",
             "sex",
             "ECOGB"
             )]<- 2 

#2. Set up the two-level structure for imputing QoL
meth <- init$method
meth[which(meth != "QoL")] <- ""
meth["QoL"]<- "2l.norm"

#3. Run the multilevel imputation and check for convergence visually
QoL_mids8 <- mice(QoL_grid, predictorMatrix = pred, method = meth, m=n.imp, maxit=1)

QoL_mids8$loggedEvents

ggmice(QoL_mids8, aes(x = QoL, group = .imp)) +
  geom_density()+
  xlab("QoL")+
  xlim(c(-20, 120))+
  theme(legend.position = c(0.25, 0.8))
ggsave(filename = "imp_density_mice_2lnorm.jpg", height = 4, width = 5.33)

QoL_mids8_long <- complete(QoL_mids8, action="long")

#4. Analysis of mean QoL while alive using a GEE with independence correlation structure
means_QoL_mids8 <- with(QoL_mids8, geeglm(QoL~as.factor(cycleno)-1, id=patid ,
                                          corstr = "independence"))
mids8_pooled <- summary(pool(means_QoL_mids8))

#------------------MICE miceadds 2l.pmm method--------------------------

#1. Initialization and setting the predictor matrix
pred <- init$predictorMatrix
pred[]<- 0
pred["QoL","patid_num"]<- -2
pred["QoL",c(paste0("cycleno_sp.", 1:N_spline_columns),
  
             "died", 
             #paste0("cyc_death_sp.", 1:N_spline_columns),
             paste0("diedxdeath.sp.", 1:N_spline_columns),

             paste0("cyc_TD_sp.", 1:N_spline_columns),
             "TD_yet",
             
             "PD", 
             "PD_yet",
             "PD_yetxPD",
             #paste0("cyc_PD_sp.", 1:N_spline_columns),
             paste0("PDxPD.sp.", 1:N_spline_columns),
             
             "Age",
             "sex",
             "ECOGB"
             )]<- 1 

#2. Set up the two-level structure for imputing QoL
meth <- init$method
meth[which(meth != "QoL")] <- ""
meth["QoL"]<- "2l.pmm"

#3. Run the multilevel imputation and check for convergence visually
QoL_mids15 <- mice(QoL_grid, predictorMatrix = pred, method = meth, m=n.imp, maxit=1, ridge=10^(-4))

ggmice(QoL_mids15, aes(x = QoL, group = .imp)) +
  geom_density()+
  xlab("QoL")+
  xlim(c(-20, 120))+
  theme(legend.position = c(0.25, 0.8))
ggsave(filename = "imp_density_mice_2lpmm.jpg", height = 4, width = 5.33)

#4. Analysis of mean QoL while alive using a GEE with independence correlation structure
means_QoL_mids15 <- with(QoL_mids15, geeglm(QoL~as.factor(cycleno)-1, id=patid ,corstr = "independence"))
mids15_pooled <- summary(pool(means_QoL_mids15))
#-----------------------------------------------
```

## Multiple imputation from a linear mixed model

```
#1. Fit an LMM to the data
imp_LMM5 <- lmer(QoL ~ (1|patid) + rms::rcs(cycleno) + rms::rcs(cycles_till_PD,nk=4, knots = c(1, 4,9,20)) + rms::rcs(cycles_till_TD,nk=4, knots = c(1,4, 9,20)) + rms::rcs(cycles_till_end,nk=4, knots = c(1,4, 9,20))*died + PD_yet + TD_yet + Age + sex + ECOGB,
                   data=QoL_grid)


#2. Drawing multiple imputations from the (approximated) posterior predictive distribution
set.seed(163)
QoL_grid.imp.LMM <- QoL_grid[,c("QoL", "patid", "cycleno", "cycles_till_PD", "cycles_till_TD", "cycles_till_end", "died", "PD", "PD_yet","TD_yet","Age", "sex", "ECOGB", "SMOKING")]

QoL_grid.imp.LMM$.id <- 1:nrow(QoL_grid.imp.LMM)
QoL_grid.imp.LMM <- do.call("rbind", replicate(n.imp+1, QoL_grid.imp.LMM, simplify = FALSE))
QoL_grid.imp.LMM$.imp <- rep(0:n.imp, each=nrow(QoL_grid))
summary(as.factor(QoL_grid.imp.LMM$.id))

QoL_grid_small <- QoL_grid[,c("QoL", "patid", "cycleno", "cycles_till_PD", "cycles_till_TD", "cycles_till_end", "died", "PD", "PD_yet","TD_yet","Age", "sex", "ECOGB", "SMOKING")]
pred.dist <- predictInterval(merMod=imp_LMM5,
                           newdata=QoL_grid_small,
                           which = "full",
                           level = 0.999,
                           n.sims = n.imp+1,
                           stat = "mean",
                           type = "linear.prediction",
                           include.resid.var = TRUE,
                           returnSims = TRUE)
sims <- (attr(pred.dist, "sim.results"))
sims_stacked <- data.frame(imp.cand = unlist(as.data.frame(sims)))
QoL_grid.imp.LMM$QoL[which(is.na(QoL_grid.imp.LMM$QoL & QoL_grid.imp.LMM$.imp>0))] <- sims_stacked$imp.cand[which(is.na(QoL_grid.imp.LMM$QoL & QoL_grid.imp.LMM$.imp>0))]

#3. Analysis of mean QoL while alive using a GEE with independence correlation structure
mids_LMM <- as.mids(QoL_grid.imp.LMM)
mids_LMM_pooled <- summary(pool(with(mids_LMM, geeglm(QoL~as.factor(cycleno)-1, id=patid ,corstr = "independence"))))
ggmice(mids_LMM, aes(x = QoL, group = .imp)) +
  geom_density()+
  xlab("QoL")+
  xlim(c(-20, 120))+
  theme(legend.position = c(0.25, 0.8))
ggsave(filename = "imp_density_LMM_mi.jpg", height = 4, width = 5.33)


#---------------------------------------------
#What if ICEs were not accounted for in the imputation?

#1. Fit an LMM imputation model that does not account for ICEs
require(lme4)
imp_LMM7 <- lmer(QoL ~ (1|patid) + rms::rcs(cycleno) + sex + Age + ECOGB, data=QoL_grid)

#2. Drawing multiple imputations from the (approximated) posterior predictive distribution
set.seed(163)
QoL_grid.imp.LMM2 <- QoL_grid[,c("QoL", "patid", "cycleno", "cycles_till_PD", "cycles_till_TD", "cycles_till_end", "died", "PD", "PD_yet","TD_yet","Age", "sex", "ECOGB", "SMOKING")]

QoL_grid.imp.LMM2$.id <- 1:nrow(QoL_grid.imp.LMM2)
QoL_grid.imp.LMM2 <- do.call("rbind", replicate(n.imp+1, QoL_grid.imp.LMM2, simplify = FALSE))
QoL_grid.imp.LMM2$.imp <- rep(0:n.imp, each=nrow(QoL_grid))
summary(as.factor(QoL_grid.imp.LMM2$.id))


pred.dist <- predictInterval(merMod=imp_LMM7,
                           newdata=QoL_grid_small,
                           which = "full",
                           level = 0.999,
                           n.sims = n.imp+1,
                           stat = "mean",
                           type = "linear.prediction",
                           include.resid.var = TRUE,
                           returnSims = TRUE)
sims <- (attr(pred.dist, "sim.results"))
sims_stacked <- data.frame(imp.cand = unlist(as.data.frame(sims)))
QoL_grid.imp.LMM2$QoL[which(is.na(QoL_grid.imp.LMM2$QoL & QoL_grid.imp.LMM2$.imp>0))] <- sims_stacked$imp.cand[which(is.na(QoL_grid.imp.LMM2$QoL & QoL_grid.imp.LMM2$.imp>0))]

#3. Analysis of mean QoL while alive using a GEE with independence correlation structure
mids_LMM_noICE <- as.mids(QoL_grid.imp.LMM2)
mids_LMM_noICE_pooled <- summary(pool(with(mids_LMM_noICE, geeglm(QoL~as.factor(cycleno)-1, id=patid ,corstr = "independence"))))
```

# Alternative: GEE weighted by the inverse probability of completeness

```
QoL_grid$complete <- !is.na(QoL_grid$QoL)
p_miss_total <- sum(QoL_grid$complete)/nrow(QoL_grid)

IPW_mod <- glm(complete ~ rms::rcs(cycleno) + rms::rcs(cycles_till_PD,nk=4, knots = c(1,4,9,20)) + rms::rcs(cycles_till_TD,nk=4, knots = c(1,4, 9,20)) + rms::rcs(cycles_till_end,nk=4, knots = c(1,4, 9,20))*died + PD + PD_yet + PD_yetxPD+ TD_yet + Age + sex + ECOGB,
                    data=QoL_grid, family = binomial(link = "logit"))
QoL_grid$pr_complete <- predict(IPW_mod, newdata = QoL_grid, type = "response")
QoL_grid$IPW_weights <- 1/QoL_grid$pr_complete
summary(QoL_grid$IPW_weights)
QoL_grid$IPW_weights_stab <- p_miss_total/QoL_grid$pr_complete
summary(QoL_grid$IPW_weights_stab)
QoL_grid$IPW_weights_trunc <- pmin(QoL_grid$IPW_weights_stab, 10)

summary(QoL_grid$IPW_weights_trunc[which(QoL_grid$cycleno==1)])

weight_GEE <- summary(geeglm(QoL~as.factor(cycleno)-1, id=patid ,corstr = "independence", data = QoL_grid, weights = IPW_weights_trunc))

aggregate(IPW_weights_stab ~ cycleno, data = QoL_grid, FUN = "max")
```

# Visualizing the results

```
#------PLOT-------------
load("imp_results.Rdata")

impstrategies.dat <- data.frame(cycleno = 1:40, 
                                  QoL_mean = obsmeans_grid$QoL[1:40],
                                  QoL_mean_SE = obsmeans_SE_grid$QoL[1:40],
                                  Model=rep("0. mean global QoL per cycle in available data", 40))

impstrategies.dat <- rbind(impstrategies.dat,
                           data.frame(cycleno = 1:40,
                                  QoL_mean = mids8_pooled$estimate,
                                  QoL_mean_SE = mids8_pooled$std.error,
                                  Model=rep("1. mice 2l.norm bsplines, RE for all variables", 40)))


impstrategies.dat <- rbind(impstrategies.dat,
                           data.frame(cycleno = 1:40,
                                  QoL_mean = mids15_pooled$estimate,
                                  QoL_mean_SE = mids15_pooled$std.error,
                                  Model=rep("2. mice 2l.pmm bsplines, random intercept", 40)))

impstrategies.dat <- rbind(impstrategies.dat,
                           data.frame(cycleno = 1:40,
                                  QoL_mean = aReg.mids_norm_pooled$estimate,
                                  QoL_mean_SE = aReg.mids_norm_pooled$std.error,
                                  Model=rep("4. aregImpute (MI) GAM rcsplines, clustered bootstrap", 40)))

impstrategies.dat <- rbind(impstrategies.dat,
                           data.frame(cycleno = 1:40,
                                  QoL_mean = aReg.mids_pmm_pooled$estimate,
                                  QoL_mean_SE = aReg.mids_pmm_pooled$std.error,
                                  Model=rep("5. aregImpute (MI) GAM pmm rcsplines, clustered bootstrap", 40)))

impstrategies.dat <- rbind(impstrategies.dat,
                           data.frame(cycleno = 1:40,
                                      QoL_mean = mids_LMM_pooled$estimate,
                                      QoL_mean_SE = mids_LMM_pooled$std.error,
                                      Model=rep("3. LMM (MI approx) rcsplines, random intercept", 40)
                        ))

impstrategies.dat <- rbind(impstrategies.dat,
                           data.frame(cycleno = 1:40,
                                  QoL_mean = weight_GEE$coefficients$Estimate,
                                  QoL_mean_SE = weight_GEE$coefficients$Std.err,
                                  Model=rep("6. IPW-GEE, log reg weights truncated at 10", 40)))

impstrategies.dat <- rbind(impstrategies.dat,
                           data.frame(cycleno = 1:40,
                                      QoL_mean = mids_LMM_noICE_pooled$estimate,
                                      QoL_mean_SE = mids_LMM_noICE_pooled$std.error,
                                      Model=rep("7. LMM (MI approx), death/PD/TD not in imputation model", 40)
                        ))


impstrategies.dat$CI_upp <- impstrategies.dat$QoL_mean+1.96*impstrategies.dat$QoL_mean_SE
impstrategies.dat$CI_low <- impstrategies.dat$QoL_mean-1.96*impstrategies.dat$QoL_mean_SE

#save(impstrategies.dat, file = "imp_results.Rdata")
#load("imp_results.Rdata")
# Load survival and available data estimates (for code see our previous paper
# entitled "The role of the estimand framework in the analysis of 
# patient-reported outcomes in single-arm trials: a case study in oncology")
load("survival_tab.Rdata")
load("avail_tab.Rdata")

impstrat <- ggplot(data = impstrategies.dat[which(impstrategies.dat$cycleno %in% c(1,6,11,16,21,26,31)),], aes(x=cycleno, y=QoL_mean, color=Model, fill=Model))+
  geom_point(alpha=0.9, position=position_dodge(3))+
  geom_errorbar(aes(ymin=CI_low, ymax=CI_upp),
                 position=position_dodge(3), width=.5)+
  ylim(c(40,80))+
  scale_x_continuous(breaks=c(1,6,11,16,21,26,31))+
  xlab("cycle number")+
  ylab("estimated mean (95% CI) global QoL while alive")+
  #ggtitle("Estimated mean global QoL [95%CI] for different imputation methods", subtitle = "While alive")+
  theme_classic()
ggarrange(impstrat, p3_till30, p4_till30, ncol = 1, heights = c(14,1,1), align = "v")
ggsave(filename = "impstrategies_compare_SEs.jpg", width = 10, height = 5)


impstrat <- ggplot(data = impstrategies.dat, aes(x=cycleno, y=QoL_mean, color=Model, fill=Model))+
  geom_point(alpha=0.9)+
  geom_line()+
  ylim(c(40,80))+
  xlab("cycle number")+
  ylab("estimated mean global QoL while alive")+
  #ggtitle("Mean global QoL per cycle for different imputation methods", subtitle = "While alive")+
  theme_bw()
ggarrange(impstrat, p3, p4, ncol = 1, heights = c(14,1,1), align = "v")
ggsave(filename = "impstrategies_compare_lines.jpg", width = 10, height = 5)

#-----------------------
```
